# Human Thymic Epithelial Cells in Serum-Free Culture: Nature and Effects on Thymocyte Cell Lines

**DOI:** 10.1155/1992/95098

**Published:** 1992

**Authors:** Carsten Ropke, Jette Elbroend

**Affiliations:** 1 Department of Medical Anatomy A University of Copenhagen The Panum Institute Copenhagen Denmark; 2 Laboratory for Cellular Immunology, Department of Medical Anatomy University of Copenhagen The Panum Institute Blegdamsvej 3 Copenhagen N DK-2200 Denmark

**Keywords:** Serum-free culture, human thymus epithelium, human thymocytes, thymocyte-epithelium interaction

## Abstract

Thymic epithelial cells (TEC) have been cultured for several months and/or for 4 to 5 transfers in a growth factor-defined serum-free medium without concurrent growth of other cell types. The use of monoclonal antibodies and *α*MAM-6 indicated that the majority of TEC were of medullary origin. The vast majority of cells were positive for LFA-3 and class I, and class II expression, was low or absent. Supernatants from the cultures were shown to contain IL-1*ß*, IL-6, and M-CSF. Coculture of cloned subpopulations of thymocytes and TEC showed effects of TEC and of secreted ILs on thymocyte proliferation. High percentages of TEC were able to bind DN, DP, or SP thymocyte populations, partly via CD2-LFA-3 adhesion. Thus, it is possible to culture TEC without unknown serum factors and with maintenance of functional activities.


*Developmental Immunology*, 1992, Vol. 2, pp. 111–121
Reprints available directly from the publisher
Photocopying permitted by license only

© 1992 Harwood Academic Publishers GmbH
Printed in the United Kingdom

### Human Thymic Epithelial Cells in Serum-Free Culture: Nature and Effects on Thymocyte Cell Lines

CARSTEN ROPKE* and JETTE ELBROEND

*Department of Medical Anatomy A, University of Copenhagen, The Panum Institute, Copenhagen, Denmark*

Thymic epithelial cells (TEC) have been cultured for several months and/or for 4 to 5 transfers in a growth factor-defined serum-free medium without concurrent growth of other cell types. The use of monoclonal antibodies and αMAM-6 indicated that the majority of TEC were of medullary origin. The vast majority of cells were positive for LFA-3 and class I, and class II expression was low or absent. Supernatants from the cultures were shown to contain IL-1β, IL-6, and M-CSF. Coculture of cloned subpopulations of thymocytes and TEC showed effects of TEC and of secreted ILs on thymocyte proliferation. High percentages of TEC were able to bind DN, DP, or SP thymocyte populations, partly via CD2-LFA-3 adhesion. Thus, it is possible to culture TEC without unknown serum factors and with maintenance of functional activities.

KEYWORDS: Serum-free culture, human thymus epithelium, human thymocytes, thymocyte-epithelium interaction.

### INTRODUCTION

The interaction between T-cell precursors and the stromal cells of the thymus is essential for induction of αβ TCR gene rearrangement and expression (e.g., Blackman et al., 1990; Ramsdell and Fowlkes, 1990; Sprent et al., 1990; von Boehmer and Kisielow, 1990). Further, stromal cells are in a key position for positive and negative selection of developing T-cell precursors. Thus, the thymic epithelial cells (TEC) of the cortex seem to select positively via MHC-TCR-peptide interaction, and negative selection is ascribed to bone marrow-derived macrophages/dendritic cells and tolerance induction to effects of medullary TEC on more mature single-positive (SP) thymocytes (reviewed by Blackman et al., 1990; Boyd and Hugo, 1991). In addition, a variety of interleukins (IL), likely to be of importance to T-cell precursor maturation, is known to be secreted by TEC, as reviewed by Haynes (1990).

To understand the role of TEC in the development of the T-cell repertoire, the nature of the direct interaction between T-cell precursors and TEC, and the importance of secreted factors (hormones/peptides), it seems essential to study well-defined cell populations in culture. Several methods have been used to culture TEC from humans (Sun et al., 1984; Berrih et al., 1985; Blue et al., 1985; Singer et al., 1985; Schuurman et al., 1986; Mizutani et al., 1987; Singer and Haynes, 1987; Galy et al., 1989). In all these reports, fetal calf serum (FCS) or human serum has been used as a necessary constituent of the culture medium. However, it is well known that the addition of serum to the medium has several disadvantages (see also Barnes and Sato, 1980), such as addition of unknown factors (hormones, antibodies, endotoxins, viruses, and other macromolecules), which may vary from batch to batch. This makes it difficult to isolate and interpret the significance of biological activities executed by the cells and by molecules released by the cells into the culture medium. The culture of TE cells in serum-containing medium is further hampered by the ability of the medium to support the growth of macrophages and fibroblasts, the former being critical in an evaluation of T-cell selection, the latter often rapidly overgrowing the epithelial cells. Although several of the previously cited papers advise methods to obtain exclusive growth of TEC, we have not succeeded in making

*Corresponding author. Present address: Laboratory for Cellular Immunology, Department of Medical Anatomy, University of Copenhagen, The Panum Institute, Blegdamsvej 3, DK-2200 Copenhagen N, Denmark.

cultures in serum-containing medium that were solely constituted by TE cells (unpublished results; Ropke et al., 1990). In the present paper, we have investigated the functional and phenotypical nature of human TEC grown in serum-free culture in the presence of well-defined growth factors. The results indicate that the cultured TEC are (1) predominantly of the medullary type, (2) bind cells of various thymocyte subsets via LFA-3/CD2 adhesion and affect their proliferation, and (3) secrete IL-1, IL-6, and M-CSF.

### RESULTS

### Characterization of TEC in Serum-Free Culture

No significant differences were detected between primary, secondary, tertiary, or quarternary cultures at a wide range of cell densities at setup as measured by $^{3}$H-TdR incorporation and scintillation countings, whereas high cell densities were required for establishment of cultures at the fifth transfer. These latter cultures usually showed a low proliferation rate (not shown). By the use of an antibody against cytokeratins 6 and 18 (K 717), the usual finding was that from 95% to 100% of the cultured cells were positive (Fig. 1a). Negative cells were usually freely growing cells and were most numerous at the start of primary cultures or in the first days after restart of frozen cultures. Another antibody against keratins, F 3006, showed similar results, although the staining generally was weaker (not shown). An antibody against cortical TEC (MAS 251) usually showed sparse reactivity in primary cultures. In later cultures, a higher reactivity, both in intensity and in numbers of positive cells, was present. However, the numbers of positive cells remained in all cultures below 20–30%, and another antibody against cortical epithelium (HB 214), showed no significant reaction in any culture (not shown). The numbers of cells positive for an antibody against medullary TEC (MAS 252) were also low at the start of primary cultures or after rethawing, but in contrast to the previous the numbers of positive cells increased with time and with transfer, and usually 80–95% of the cells were positive in "late" cultures (Fig. 1b). These findings indicate that the majority of the cultured cells were of medullary origin—or that cytokeratins characteristic for medullary TEC were increasingly expressed by prolonged culture.

Findings in support for this were obtained by the use of an antibody against outer cells of Hassall's bodies (MAS 256). Although a few cells were positive at the start of culture, nearly all cells were positive at the later time intervals, especially in dense cultures (not shown). Also, small accumulation of cells, which showed the features of Hassall's bodies at light microscopy of sections of the cultures (not shown), were always positive. Further, an antibody (MAM-6) against sialomucins (Hilkens et al., 1989), which has shown reactivity with secretory epithelia and Hassall's bodies (Hilkens et al., 1984), showed positive reaction on a high number of cultured cells in most cultures irrespective of their transfer number (Figs. 1c and 2). By the use of an antibody against mesenchymal cells (MAS 253), only very few positive cells were observed—below 5% These cells were always found as singles in the area between developing islands of epithelial cells and disappeared with time (not shown).

High percentages of cultured cells were positive for class I and LFA-3 antibodies, whereas the class II expression generally was low or absent. Less than 10% of the cells showed a significant reaction (Fig. 2).

### Culture of Thymocyte Cell Lines in the Presence of TEC

Frozen thymocytes from the TEC donors were thawed and set up in Terasaki plates as described in Materials and Methods. Maintenance of the cell lines was dependent on the presence of IL-2, which increased the TdR incorporation 25× after 3 days of culture. PHA further increased the incorporation 2×, and Con A depressed the incorporation of TdR in IL-2 containing cultures about 2×.

To investigate the effects of TEC on the proliferation of cloned thymocytes, the following experiments were set up partly to clarify effects on the IL-2-driven proliferation, and partly to detect a possible comitogenic effect of TEC on PHA-induced proliferation, the latter used as an indicator for functional capability of the cloned thymocytes.

Figure 3 shows the results of a typical experiment, in which variable numbers of TEC were cultured for 3 days together with a thymocyte cell line. As can be seen, a significant depression in TdR incorporation is found by a TEC percentage at and above 5 in the cultures.

Table 1 shows the results from 21 experiments, in which 19 different cell lines, derived from 3 different donors, were cultured with or without TEC from 5 different donors. As can be seen from the table, a much higher proliferation was found in cultures of thymocytes without admixed TEC than in cultures containing TEC. All ratios were below 1, the average being 0.49±0.18(SD).

In some experiments, the $^{3}$H-TdR incorporation in the cultures was measured on each of the 3 days of culture. No significant change in the ratio was found (not shown).

The table shows further that a very significant

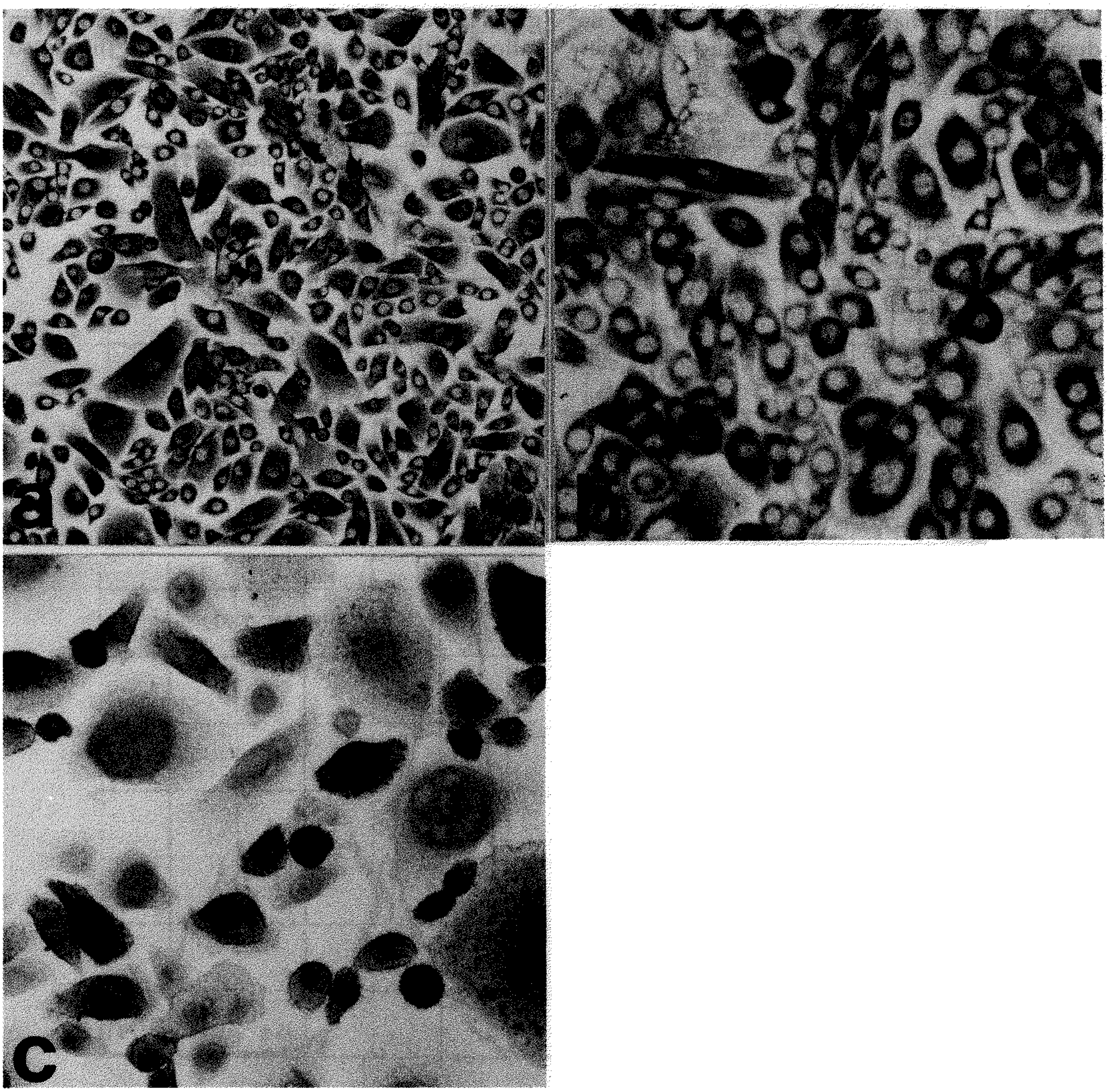

FIGURE 1. Photomicrographs of cultures of serum-free growth human thymus epithelial cells. Cells are stained by the PAP method after incubation with antibodies against (a) cytokeratins 6 and 18 (×126), (b) medullary epithelial cells (×250), and (c) surface antigens of secretory epithelial (×250).

change was found when PHA was added to the cultures. Here, a high $^3$H-TdR incorporation was found in TEC-containing cultures as compared to cultures without TEC. Thus, the ratio between the cultures was inversed when PHA was added to the cultures, the average of PHA containing cultures being 1.89±0.73 of 13 experiments.

The ratio changed less than 10% in experiments in which cloned thymocytes were grown with either irradiated (2500 rad) or nonirradiated TEC, which indicates that the previous effects were not dependent on initiation of synthesis of new secreted and/or surface-bound molecules (not shown).

Although it seems likely that the comitogenic effect of TEC on cloned thymocyte proliferation is due to a directly TEC induced stimulation, parallel to PHA stimulation, an indirect effect mediated via PHA stimulation of TEC—or PHA-induced increased aggregation of cells—cannot be excluded.

The CD antigen composition of the cell lines is given in Table 1 together with the individual cpm ratios. At the time of the experiments, the stated

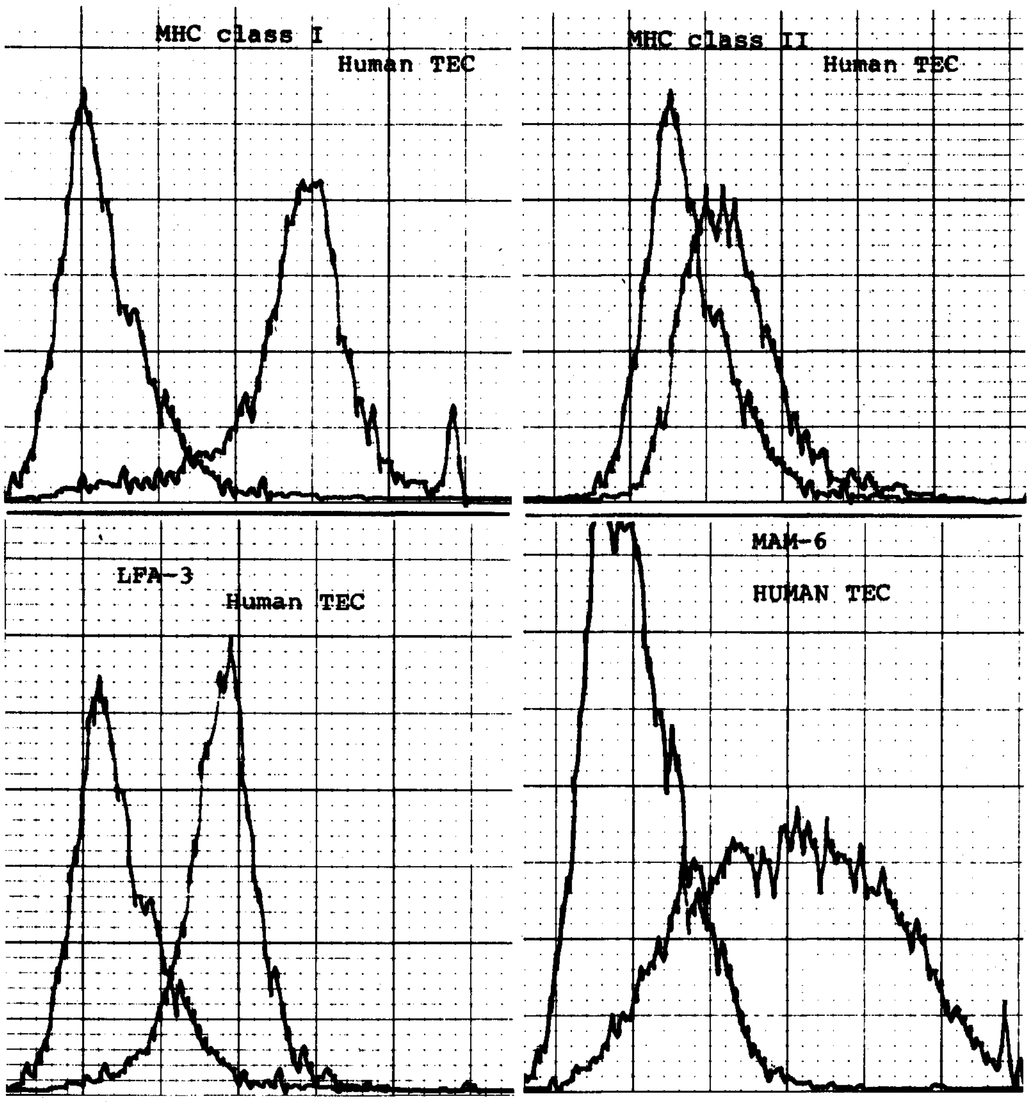

FIGURE 2. FACS histograms of serum-free grown human thymus epithelial cells (TEC) from secondary cultures. Cells were incubated with a-MHC class I, a-MHC class II, a-LFA-3, or MAM-6 (an antibody against surface antigens of secretory epithelia) antibodies, followed by incubation with FITC-labeled-GAM antiserum. Ordinate: relative cell numbers; abscissa: log fluorescence. Curves on the left-hand side show fluorescence of cells labeled with FITC-GAM only.

CD antigen composition of the lines had remained stable for at least 3 weeks, and no differences were observed between cells grown with or without TEC, or between cell lines before and after culture (not shown).

### Interleukin Secretion from Cultured TEC

Supernatants obtained from serum-free cultures of TEC from four donors were tested in an ELISA assay including anti-IL-1$\alpha$ and -IL-1$\beta$. In three of the supernatants IL-1$\beta$ was detected—2300, 5800, and 5500 pg/mL, respectively—and tests for IL-1$\alpha$ were negative (Ropke and Bendtzen, unpublished results). To test the importance of IL-1$\beta$ for the proliferation of thymocytes±TEC in the presence of PHA, IL-1$\beta$ was added to the cultures in variable amounts (Fig. 4). The figure shows that both the proliferation of thymocytes cultured alone and of thymocyte–TEC cultures was increased by addition of IL-1. The proliferation of TEC was not altered significantly by addition of IL-1 (not shown), in opposition to results reported by Galy et al. (1989). Further, the effects of preculture of TEC in the presence of IL-1 were tested and compared to the effect of direct addition of IL-1 to the cultures with or without

TABLE 1
Effects of Serum-Free Cultured Thymus Epithelium on Proliferation of Thymocyte Lines[a]

| Cell line[b] | TEC[c] | Ratio–PHA[d] | Ratio+PHA | CD 4/8%[e] |
|---|---|---|---|---|
| W 14 | Re | 0.5 | | 100/50 |
| W 4 | Re | 0.6 | 2.9 | 100/100 |
| W 8 | Re | 0.5 | 1.8 | 100/0 |
| W 26 | Re | 0.6 | 2.5 | 80/100 |
| W 34 | Re | 0.5 | 3.3 | 0/50 |
| R 1 | Ka-C | 0.7 | | 100/100 |
| R 2 | Ra | 0.4 | 2.1 | 60/0 |
| K 1 | Ra | 0.5 | 2.5 | 75/25 |
| K 3 | Hu | 0.7 | | 0/100 |
| K 5 | Hu | 0.3 | | 40/60 |
| K 9 | Hu | 0.2 | | 30/80 |
| K 11 | Hu | 0.4 | | 0/100 |
| K 13 | Hu | 0.4 | | 80/20 |
| K 16 | Hu | 0.4 | | 20/80 |
| K 2 | Ka-K | 0.4 | 1.4 | 100/0 |
| K 5 | Ka-K | 0.2 | 1.3 | 75/0 |
| K 9 | Ka-K | 0.4 | 1.1 | 25/75 |
| K 18 | Ka-K | 0.5 | 1.9 | 100/0 |
| K 19 | Ka-K | 0.6 | 1.3 | 25/75 |
| K 23 | Ka-K | 0.5 | 2.6 | 100/0 |

[a]Thymocytes were cultured for 3 days±thymic epithelial cells±PHA.
[b]Thymocyte lines derived from limiting dilution culture of thymocytes.
[c]Identity of serum-free grown thymic epithelial cells (TEC), of which 5000 were added to each culture of 50,000 cloned thymocytes, or grown alone.
[d]The ratio between average cpm of cloned thymocytes cultured for 3 days±PHA with TEC or without TEC:cpm (thymocytes+TEC)–cpm TEC/cpm thymocytes.
[e]Percentage $CD4^+CD8^+$ cells of the various cell lines.

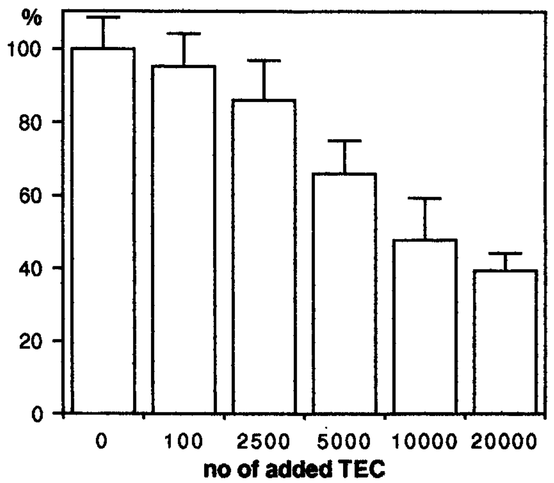

FIGURE 3. Histogram showing $^3$H-TdR incorporation in cloned $CD4^+$ thymocytes. Cultures of $10^5$ $CD4^+$ cells were grown in 3 days in the presence of IL-2 and the given numbers of thymic epithelial cells (TEC.) Results are given as percentage values±SD. The mean cpm of cultures without added TEC was set to 100. Cpm's of parallel cultures of TEC alone are subtracted from the cpm's of cultures of $CD4^+$ cells+TEC.

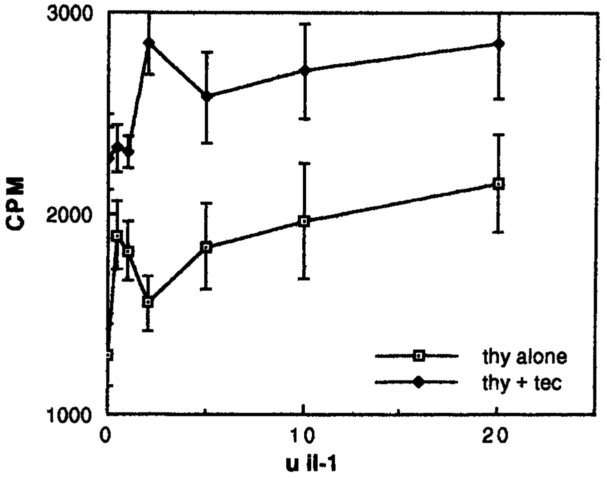

FIGURE 4. Effects of rIL-1-$\beta$ on proliferation in 3-day cultures of 50,000 cloned $CD4^+$ thymocytes in the presence of PHA. $CD4^+$ cells were either cultured alone (thy alone) or in the presence of 5000 thymic epithelial cells (thy+tec). Results are given as mean $^3$H-TdR incorporation±SD. Cpm's of parallel cultures of 5000 epithelial cells are subtracted from the results of (thy+tec) cultures.

addition or rIL-2. Figure 5 shows that, in the absence of IL-2, very high counts are obtained by PHA addition to IL-1 preincubated cultures compared with counts from other cultures, and proliferation was negligible in cultures without PHA. The high proliferation rate in cultures including IL-1 preincubated TEC, as compared to other cultures, was maintained after addition of IL-2 to PHA containing cultures, whereas—in the absence of PHA—the proliferation was diminished in TEC-containing cultures, also with respect to cultures of thymocytes alone, in agreement with the previous findings (Table 1). To test if IL-1 was working via IL-6 on thymocyte proliferation, experiments, in which αIL-6 was included in some of the cultures, were performed. The results given in Figure 6 show that the increased thymocyte proliferation induced by TEC was abolished by the addition of αIL-6 to the cultures, both with regard to "normal" TEC and TEC preincubated with IL-1. Thus, these latter experiments indicate that the beneficial effect of TEC on proliferation of cloned thymocyte mainly is mediated via secretion of IL-6, a secretion that is augmented by IL-1. Experiments, in which αLFA-3 was included in PHA-containing cultures of thymocytes±TEC, showed that the beneficial effects of TEC on thymocyte proliferation was diminished by the addition of this antibody (not shown), indicating that CD2/LFA-3 adherence (as depicted by "Rosette Formation"; see what follows) is essential for the increase in thymocyte response to PHA induced by TEC.

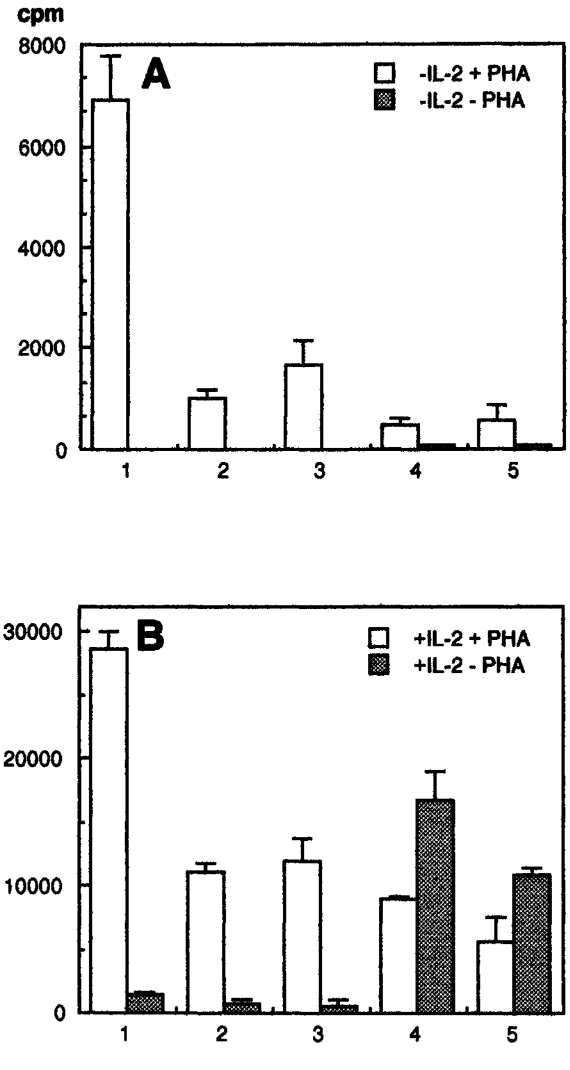

FIGURE 5. Effects of preculture for 3 days of thymic epithelial cells (tec) in the presence of rIL-1-β before coculture with cloned $CD4^+$ thymocytes as compared with cocultures of $CD4^+$ cells and not-precultured tec. Further, the effects of PHA and IL-2 on cocultures are shown. $CD4^+$ cells were either cultured without (A) or with (B) IL-2. In addition, cultures were set up both in the presence (blank columns) and absence (shaded columns) of PHA. Five types of cultures are compared: 1, $CD4^+$ cells+precultured tec; 2, $CD4^+$ cells+tec+10 μ IL-1; 3, $CD4^+$ cells+tec; 4, $CD4^+$ cells+10 μ IL-1; 5, $CD4^+$ cells. Results are given as mean cpm±SD of triplicate cultures. Cpm's of parallel cultures of tec are subtracted from the results of cultures of $CD4^+$ cells+tec

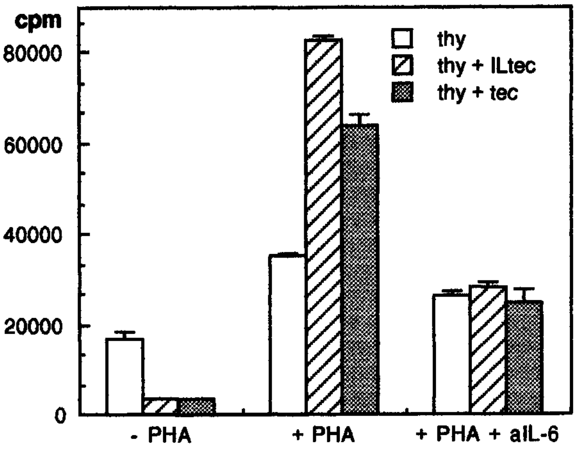

FIGURE 6. Histogram showing that anti-IL-6 antibody (aIL-6) abolishes the beneficial effects of thymic epithelial cells (tec) on the PHA-induced proliferation of cloned $CD4^+$ thymocytes. Fifty thousand cloned $CD4^+$ thymocytes (thy) were cultured for 3 days with or without 5000 thymic epithelial cells in the presence of rIL-2. Tec were either precultured for 3 days in the presence of 10 μ rIL-1β (ILtec) or used directly. To some of the cultures, anti-IL-6 antibody (aIL-6) was added at the start of the cultures. Results are given as mean cpm±SD of triplicate cultures. Cpm's of parallel cultures of tec are subtracted from the results of cultures of thy+tec.

### Secretion of M-CSF from Cultured TEC

Addition of TEC supernatant to MC cultures of murine bone marrow cells resulted in a number of developing clones after 6 days, a number that was usually between 40% to 70% of that given by the same percentage of supernatant from Con-A stimulated spleen cells (not shown). High numbers of colonies developed in the MC after addition of cultured TEC directly to the BM cell cultures. Figure 7 shows a typical experiment, in which increasing numbers of TEC were added, and the figure shows that the optimal stimulation of colony formation was obtained after addition of about 20,000 TEC to 100,000 BM cells. Addition of IL-1$\beta$ to the cultures about doubled the numbers of colonies induced by TEC (not shown). Cell morphology after orcein staining of colonies indicated that only macrophage colonies developed in the cultures, pointing out that only M-CSF was secreted by the TEC.

### Rosette Formation between TEC and Thymocytes

When thymocytes are incubated with TEC, a number of TEC will bind thymocytes in rather stable aggregates—rosettes—both at 4 °C and 37 °C. These rosettes can be counted by light microscopy. Table 2 shows results from such experiments in which TEC from seven donors and 24 cell lines from five donors were set up. As can be seen from the table, no significant differences were found between different CD subpopulations in percent rosette formation, all showing a rather high percentage at 4 °C, whereas the two subpopulations tested at 37 °C showed lower percentages. The effects of addition of antibodies against surface molecules to the cultures were evaluated by the use of $CD4^+$ cell line. As can be seen from the table, interaction between CD2 and LFA-3 seems to be essential for binding of thymocytes to TEC at 4 °C. No significant differences in rosette formation were found when a cell line was incubated with TEC from different donors including TEC from the donor of the cell line (not shown).

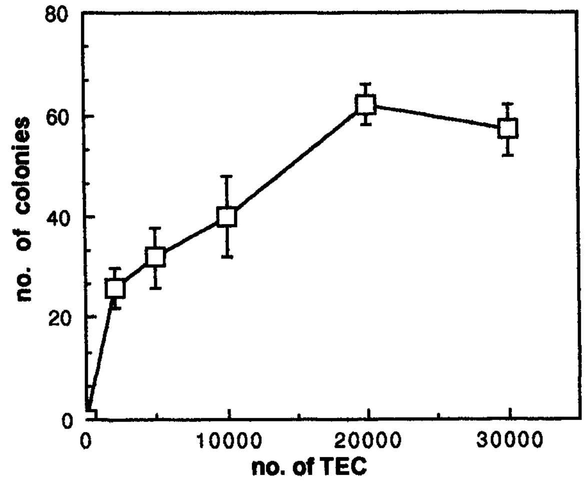

FIGURE 7. Number of murine bone marrow colonies developing in 1 mL methylcellulose cultures of 100,000 bone marrow cells after addition of variable numbers of serum-free grown thymus epithelial cells (TEC). Means±SD of triplicate cultures are given.

### DISCUSSION

By the use of the present culture method, it is possible to grow TEC without any appreciable growth of mesenchymal cells, and without any additional separation procedures, as judged from the results obtained with antibodies against cytokeratins. These results indicate further that the majority of cultured TEC belong to a population of secretory cells derived from the thymic medulla, although some TEC with cortical phenotype were regularly found. Results from culture of

TABLE 2
Binding of Cloned Subsets of Thymocytes to Serum-Free Grown TEC[a]

| Phenotype of cell line | Additive[b] | Temperature[c] 4 °C | Temperature[c] 37 °C | % Depression[e] | No. of exp. |
|---|---|---|---|---|---|
| $CD4^+8^+$ | | 61±20[d] | 11±10 | | 9 |
| $CD4^-8^+$ | | 63±22 | | | 7 |
| $CD4^+8^+$ | | 43±8 | 5±4 | | 4 |
| $CD4^-8^-$ | | 46±26 | | | 5 |
| $CD4^+8^-$ | αCD4 | | | 0 | 4 |
| | αCD3 | | | 0 | 3 |
| | αCD2 | | | 50±22 | 6 |
| | αLFA-3 | | | 44±23 | 6 |
| | αCD2+ αLFA-3 | | | 72±14 | 5 |

[a]Thymus epithelial cells (TEC) were incubated with cloned thymocytes of the given phenotypes for 1/2 h in the ratio 1 to 10.
[b]Antibodies added to cultures.
[c]Cells were incubated at the given temperatures.
[d]Percentage of TEC with bound thymocytes±SD.
[e]Percentage of thymocyte binding TEC in incubations without added antibodies was set to 0.

human TEC in a serum-containing medium point to a mixed cortical/medullary origin of the cultured TEC (Schuurman et al., 1986; Singer and Haynes, 1987), but cultured cells of subcapsular origin have also been reported (Mizutani et al., 1987). Because of the separate functions in the selection of T-cell clones assigned to cortical and medullary TEC, respectively, as surveyed by Ramsdell and Fowlkes (1990), Sprent et al. (1990), von Boehmer and Kisielow (1990), and Boyd and Hugo (1991), it is of major importance in the evaluation of the T-cell progenitor—TEC interactions in vitro to clarify the nature of the cultured TEC. The present results, indicating predominance of medullary TEC, may show that the culture medium is primarily supportive for medullary TEC. However, to our knowledge, no information on possible phenotypic shifts of TEC brought from in vivo to in vitro conditions is reported. Further, in spite of the generally accepted view that medullary and subcapsular TEC are of ectodermal origin and cortical TEC of endodermal origin (Haynes, 1990), there is no conclusive evidence for this view. Thus, it is possible that cells with potentials for differentiation into both cortical and medullary phenotypes/functions are present in the cultures and may differentiate in a given direction depending upon the culture conditions (e.g., presence of ILs or thymocytes).

The serum-free cultured TEC of the present study show significant amounts of class I and LFA-3 molecules, but only a limited expression of class II molecules, in accordance with findings in serum-containing cultures, although addition of interferon-$\gamma$ or thymocytes to the cultures augments the amount of class II molecules on TEC (Berrih et al., 1985; Denning et al., 1987). This has not been tested in the present study, but we have shown, by the use of several cell lines derived from different populations of thymocytes, that the cultured TEC affect the IL-2-dependent proliferation of these populations, resulting in a depression in $^{3}$H-TdR incorporation in the absence of mitogen, whereas TEC augmented the PHA response of the cell lines. This reverse effect seems to indicate that several receptors are involved in the interaction between the cells. Thus, both ICAM-1 and LFA-1 interaction as well as LFA-3 and CD2 interaction are involved in thymocyte-TEC adhesion as shown by rosette formation (Vollger et al., 1987; Nonoyama et al., 1989; Singer et al., 1990; present study). The adhesion is likely to be an essential step for further interactions, and has previously been shown to be characteristic for early thymocytes expressing rather low amounts of CD3 (Nonoyama et al., 1989). However, in the present study, we show that high percentages of TEC bind both early and late thymocyte subsets, the latter showing CD3 expression comparable to peripheral T cells (not shown). The difference between these studies may be due to that we have tested actively proliferating cell lines rather than G0/1 cells, our results being in accordance with those of Singer et al. (1990), who tested Con A-stimulated cells.

Several studies performed with serum-containing cultures have shown that cultured TEC secrete a number of interleukins: IL-1, IL-6, TNF, LIF, and GM-CSF (Le et al., 1987; Galy et al., 1990; Le et al., 1990) of possible importance to the development and differentiation of T-cell precursors. In the present serum-free cultures, we have so far obtained evidence for secretion of some of these ILs and of effects on T-cell lines of these ILs. Our results indicate that at least a part of the IL-1$\beta$ effect is mediated through stimulation of TEC to secrete IL-6, which acts on the thymocyte subsets as demonstrated by Galy et al. (1990). Further studies of serum-free cultures are needed to elucidate the constitutive secretion of cultured TEC. This applies also to the present demonstration of secretion by TEC of M-CSF but not GM-CSF, the latter being demonstrated in serum-containing cultures (Galy et al., 1990; Le et al., 1990). Low amounts of GM-CSF may result in formation of macrophage colonies only (Metcalf, 1977), but the presence of rather high numbers of colonies (Fig. 7) and high numbers of cells in the individual colonies (unpublished results) do not indicate that this is the reason for the lack of demonstrable GM-CSF.

In conclusion, the present results indicate that the serum-free culture of TEC allows for study of the T-cell precursor–TEC interactions under well-defined conditions as well as a dissection of the pedigree of IL effects in T-cell development. However, we have not in the present study—which was focused on cultures consisting solely of TEC—been able to test whether the presented effects on thymocyte cell-line proliferation are specific for TEC. This is now being tested in our laboratory.

### MATERIALS AND METHODS

### Material

Thymic tissue was obtained from children, ages 1/2 to 2 years, undergoing cardiovascular surgery for congenital heart disease. The tissue was cut into approximately 1–2-mm fragments by the use of surgical knives, and fragments were washed several times in PBS. The washing medium, including the free cells, was collected and the free cells were frozen in liquid nitrogen after addition of 10% DMSO to the medium for later establishment of thymocyte cell lines. The tissue fragments were incubated for 1 h at 37 °C in medium containing 5.0 mg/mL DNAase (Sigma, St. Louis, MO) and 1.5 mg/mL collagenase/dispase (Boehringer, Mannheim, FRG) in a culture dish, which was shaken regularly. Thereafter, the sizes of the fragments were considerably below 1 $mm^3$. The fragments were washed two times with the medium before cultivation.

### Culture Medium and Additives

The serum-free, hormonally defined growth medium used was composed of a 1:1 mixture (v/v) of Ham's F12 medium and Dulbecco's modified Eagle medium (DMEM) (Flow Laboratories, Hillerød, Denmark). The medium was supplemented with 2 mM Glutamine (Sigma, G 3126), 250 IU penicillin/mL, 25 mg streptomycine/mL, 3 mg/mL insulin (0.025 IU/mg, Nordisk Insulin, Denmark), 100 ng/mL epidermal growth factor, 0.5 mg/mL hydrocortisone (both from Collaborative Research, USA), and 10 ng/mL cholera toxin (Sigma).

### TEC Culture

The cells were cultured in 5 mL medium in T-25 flasks, or in 1 mL medium in two-chamber Lab-Tec Chamber slides (both NUNC, Roskilde, Denmark). All containers for culture were coated with type-I collagen, 8 mg/$cm^2$ (Vitrogen-100; Flow Laboratories). Cultures were maintained in a humidified atmosphere of 5% $CO_2$ with 20% $O_2$ and 75% $N_2$. The medium was changed every 2–3 days. At cell transfer, TE cells were detached from the plastic by treatment with trypsin/EDTA.

### Cloning of Thymocytes

After thawing, living cells were recovered from the frozen thymocyte suspensions through a Percoll cushion, washed twice, and cloned by limiting dilution (0.3 cells/well) in Terasaki plates (NUNC) in 20 $\mu$L RPMI 1640 medium (Gibco Laboratories, Grand Island, NY), supplemented with 5% human serum, 2 mM L-glutamine, 5 $\mu$L Phytohemagglutinine (PHA, Wellcome, Beckenham, Kent, UK), 100 U/mL human rIL-2 and 5× $10^5$/mL 2500 rad irradiated allogeneic PBMC as feeders, as adapted from De Libero and Lanzavecchia (1989). After 8–10 days positive wells were scored and cells were further grown as before.

### Immunocytochemistry and Antibodies

Cytokeratins were detected with DAKO-CK1 (M 717) specific to keratins 6 and 18 (DAKOPATTS, Glostrup, Denmark) and with MON F 3006 (SANBIO, Uden, The Netherlands), an antibody against keratin 18. MAM-6, a monoclonal antibody against human epithelial sialomucins (Hilkens et al., 1989), was used for detection of secretory epithelium (gift from J. Hilkens, The Netherlands Cancer Institute, Amsterdam). The monoclonals MAS 251, 252, 253, and 256 (Sera-Lab, Sussex, UK) against cortical TEC, subcapsular and medullary TEC, mesenchymal cells, and cells in outer swirls of Hassall's bodies, respectively, and HB 214–anticortical epithelium (ATTC) were used for detection of these cells. The second antibody used was a rabbit polyclonal antibody against mouse immunoglobulins (259, DAKOPATTS), and the third reagent was a monoclonal peroxidase antiperoxidase (PAP) complex (P850, DAKOPATTS). The cytochemical incubation was performed as described by Petersen and van Deurs (1988). Fluorescein (FITC), Phycoerythrin (PE) and biotin conjugates of monoclonal antibodies against CD4 and CD8 were purchased from Becton Dickinson (Sunnyvale, CA). Monoclonals against CD2 and CD3 (110.08 and 101.01) were prepared and given by Dr. T. Plesner (Gentofte County Hospital, Copenhagen). Anti-LFA-3, anti-MHC class I, and class-II antibodies were obtained from ATCC (HB 205, HB 95, and HB 104). Specific rabbit antiserum to human IL-6 (Hansen et al., 1991) was given by Dr. K. Bendtzen (Rigshospitalet University Hospital, Copenhagen).

### Fluorescence Labeling and Registration of Cells

Cells to be analyzed in the FACS or by fluorescence microscopy were incubated with the appropriate amount of FITC-, PE-, or biotin-labeled antibodies in an ice bath for 30 min. In the case of biotin labeling, cells were incubated with avidin-PE (Becton Dickinson) for 30 more minutes after a wash. Cells were washed two times in phosphate-buffered saline (PBS) containing 1% fetal calf serum (FCS, GIBCO) before use. A FACS III equipped with logarithmic amplifiers for the fluorescence signals and dual-fluorescence compensation was used. The setup has previously been described (Ropke, 1984). A Leitz Ortholux II epifluorescence microscope was used for fluorescence microscopy.

### Interleukins

Recombinant IL-1$\beta$ (rIL-1$\beta$) (Dalbølge et al., 1989) was a gift from K. Hejnaes (Hagedorn Research Laboratory, Copenhagen, Denmark). IL-1 activity was tested in a murine thymocyte assay according to Le et al. (1987). RIL-2 (Proleukin) was obtained from EuroCetus B.V. (Amsterdam, Holland).

### Culture of Thymocyte Cell Lines±TEC

Cell lines were cultured for 72 hr in RPMI-1640 in the presence of 100 u/mL rIL-2 and 5% FACS in round-bottom multidishes (NUNC). The number of thymocytes was 50,000 per well, and the numbers of TEC 1/10 of the thymocyte number. Results are expressed as the ratio between $^3$H-Thymidine ($^3$H-TdR) incorporation in cultured of thymocytes+TEC–$^3$H-TdR incorporation in cultures of TEC alone/$^3$H-TdR incorporation in cultures of thymocytes alone: cpm (thy+TEC)–cpm TEC/cpm thy. $^3$H-TdR, specific activity 22 Ci/mM (N.E.N., Boston, MA), 2.5 mCi/mL culture, was added to cultures 6 h before harvest.

### Detection of CSF

Cells were cultured in 33-mm plastic dishes (NUNC) in 1 mL 0.96% MC (Fluca, Buchs, Switzerland) in Dulbecco's modified Eagle's medium (GIBCO). The medium was supplemented with 5×10$^5$ M 2-mercaptoethanol (Fluca), 12% FCS, 4% horse serum (Statens seruminstitut, Copenhagen, Denmark), 5 $\mu$L/mL PHA, and variable percentages of TEC or TEC supernatants (see Results). In control cultures, 10% spleen-conditioned medium, which was obtained as supernatant from 10×10$^6$ rat spleen cells cultured for 24 h at 37 °C in RPMI-1640 medium including 5% FCS and 4 $\mu$g/mL Concanavalin A (Pharmacia, Uppsala, Sweden), was used as a CSF source. Developing colonies (cell aggregates >50 cells) were counted in the MC by means of a stereomicroscope at a magnification of ×20 to ×40 on day 6 of culture.

Morphology of the colony cells was detected by light microscopy after orcein staining of single dried colonies.

### Rosette Formation between TEC and Thymocytes

Rosettes—aggregates of a TEC with three or more bound thymocytes after coincubation of the cells at 4 °C or 37 °C—were performed according to Singer et al. (1990). The ratio TEC/thymocytes was 1/10. In the case of antibody addition to the cultures, these were added at the start of the incubation.

### ACKNOWLEDGMENTS

We thank Ane-Marie Rulykke for excellent technical assistance and Keld Ottosen for expert photographical assistance. Dr. Poul Lauridsen and his staff, Department of Thorax Surgery, Rigshospitalet, Copenhagen, are thanked for their generous gifts of thymus specimens. This work has been supported by the Danish Research Council, the NOVO Foundation, the P. Carl Pedersen Foundation, and the C. L. David Foundation.

*(Received May 14, 1991)*

*(Accepted September 25, 1991)*

### REFERENCES

Barnes D.W., and Sato G.H. (1980). Serum-free culture: A unifying approach. Cell **22:** 649–655.

Berrih H., Savino W., and Cohen S. (1985). Extracellular matrix of the human thymus; immunofluorescence studies on frozen sections and cultured epithelial cells. J. Histochem. Cytochem. **33:** 655–664.

Berrih S., Arenzana-Selsoedos F., Cohen S., Devos R., Charron D., and Virelizler I.J. (1985). Interferon-$\gamma$ modulates HLA Class II antigen expression on cultured thymic epithelial cells. J. Immunol. **135:** 1165–1171.

Blackman M., Kappler J., and Marrack P. (1990). The role of the T cell receptor in positive and negative selection of developing T cells. Science **248:** 1335–1341.

Blue M.-L., Daley J.F., Levine H., and Schlossman S.F. (1985). Class II major histocompatibility complex molecules regulate the development of the $T4^+T8^-$ inducer phenotype of cultured human thymocytes. Proc. Natl. Acad. Sci. USA **82:** 8178–8182.

Boyd R.L., and Hugo P. (1991). Towards an integrated view of thymopoiesis. Immunol. Today **12:** 71–79.

Dalbøge H., Bayne S., Christensen T., and Heljnaes K.R. (1989). Cloning and expression of an interleukin-1$\beta$ precursor and its conversion to IL-1$\beta'$. FEBS Lett. **246:** 89–93.

De Libero G., and Lanzavecchia A. (1989) Establishment of human double-positive thymocyte clones. J. Exp. Med. **170:** 303–308.

Denning S.M., Tuck D.T., Singer K.H., and Haynes B.F. (1987). Human thymic epithelial cells function as accessory cells for autologous mature thymocyte activation. J. Immunol. **138:** 680–686.

Galy A.H.M., Dinarello C.A., Kupper T.S., Kameda A., and Hadden J.W. (1990). Effects of cytokines on human thymic epthelial cells in culture. II. Recombinant IL1 stimulates thymic epithelial cells to produce IL 6 and GM-CSF. Cell Immunol. **129:** 161–175.

Galy A.H.M., Hadden E.M., Touraine J.-L., and Hadden J.W. (1989). Effects of cytokines on human thymic epithelial cells in culture: IL-1 induces thymic epithelial cell proliferation and change in morphology. Cell. Immunol. **124:** 13–27.

Hansen M.B., Svenson M., Diamant M., and Bendtzen K. (1991). Anti-interleukin-6 antibodies in normal human serum. Scand J. Immunol. **33:** 777–781.

Haynes, B.F. (1990). Human thymic epithelium and T cell development: Current issues and future directions. Thymus **16:** 143–157.

Hilkens J., Buijs F., and Ligtenberg M. (1989). Complexity of MAM-6, an epithelial sialomucin associated with carcinomas. Cancer Res. **49:** 786–793.

Hilkens J., Buijs F., Hilgers J., Hageman P., Calafat J., Sonnenberg J.A., and van der Valk M. (1984). Monoclonal antibodies against human milk-fat globule membranes detecting differentiation antigens of the mammary gland and its tumours. Int. J. Cancer **34:** 197–206.

Le P.T., Lazorick S., Whichard L.P., Yang Y.-C., Clark S.C., Haynes B.F., and Singer K.H. (1990). Human thymic epithelial cells produce IL-6, granulocyte-monocyte-CSF, and leukemia inhibitory factor. J. Immunol. **145:** 3310–3315.

Le P.T., Tuck D.T., Dinarello C.A., Haynes B.F., and Singer K.H. (1987). Human thymic epithelial cells produce interleukin 1. J. Immunol. **138:** 2520–2526.

Metcalf D. (1977). Hemopoietic colonies. In vitro cloning of normal and leukemia cells. Recent results in cancer research (Berlin: Springer-Verlag).

Mizutani S., Watt S.M., Robertson D., Hussein S., Healy L.E., Furley A.J.W., and Greaves M.F (1987). Cloning of human thymic subcapsular cortex epithelial cells with T-lymphocyte binding sites and hemopoietic growth factor activity. Proc. Natl. Acad. Sci. USA **84:** 4999–5003.

Nonoyama S., Nakayama M., Shiohara T., and Yata J.-I. (1989). Only dull $CD3^+$ thymocytes bind to thymic epithelial cells. The binding is elicited by both CD2/LFA-3 and LFA-1/ICAM-1 interactions. Eur. J. Immunol. **19:** 1631–1635.

Petersen O.W., and van Deurs B. (1988). Growth factor control of myoepithelial-cell differentiation in cultures of human mammary gland. Differentiation **39:** 197–215.

Ramsdell F., and Fowlkes B.J. (1990). Clonal deletion versus clonal energy: The role of the thymus in inducing self tolerance. Science **248:** 1342–1348.

Ropke C. (1984). Characterization of T lymphocyte colony-forming cells in the mouse. The colony-forming cell is confined to Lyt-1,2,3+ cell subsets in the thymus and the lymph nodes. J. Immunol. **132:** 1625–1631.

Ropke C., Petersen O.W., and van Deurs B. (1990). Short-term cultivation of murine thymic epithelial cells in a growth factor defined serum-free medium. In Vitro Cell. Dev. Biol. **26:** 671–681.

Schuurman H.J., Hendriks R.W., Lange J.M.A., van der Linden J.A., Gmelig Meyling F.H.H., Danner S.A., and Kater L. (1986). Cultured human thymus epithelial monolayer cells induce CD4 expression on mononuclear cells of AIDS patients in vitro. Clin Exp. Immunol. **64:** 348–355.

Singer K.H., Denning S.M., Whichard L.P., and Haynes B.F. (1990). Thymocyte LFA-1 and thymic epithelial cell ICAM-1 molecules mediate binding of activated human thymocytes to thymic epithelial cells. J. Immunol. **144:** 2931–2939.

Singer K.H., Harden E.A., Robertson A., Lobach D.F., and Haynes B.F. (1985). In vitro growth and phenotypic characterization of mesodermal-derived and epithelial components of normal and abnormal human thymus. Hum. Immunol. **13:** 161–176.

Singer K.H., and Haynes B.F. (1987). Epithelial-thymocyte interactions in human thymus. Hum. Immunol. **20:** 127–144.

Sprent J., Gao E.-K., and Webb S.R. (1990). T cell reactivity to MHC molecules: Immunity versus tolerance. Science **248:** 1357–1363.

Sun T.-T., Bonitz P., and Burns W.H. (1984). Cell culture of mammalian thymic epithelial cells: Growth, structural, and antigenic properties. Cell Immunol. **83:** 1–13.

Vollger L.W., Tuck D.T., Springer T.A., Haynes, F., and Singer K.H. (1987). Thymocyte binding to human thymic epithelial cells is inhibited by monoclonal antibodies to CD-2 and LFA-3 antigens. J. Immunol. **138:** 358–363.

von Boehmer H., and Kisielow P. (1990). Self-nonself discrimination by T cells. Science **248:** 1369–1373.

